# Variation in Antioxidant Attributes at Three Ripening Stages of Guava (*Psidium guajava* L.) Fruit from Different Geographical Regions of Pakistan

**DOI:** 10.3390/molecules17033165

**Published:** 2012-03-14

**Authors:** Javaria Gull, Bushra Sultana, Farooq Anwar, Rehana Naseer, Muhammad Ashraf, M. Ashrafuzzaman

**Affiliations:** 1 Department of Chemistry and Biochemistry, University of Agriculture Faisalabad-38040, Pakistan; Email: javeria.gull@yahoo.com (J.G.); rehanan735@gmail.com (R.N.); 2 Department of Chemistry, University of Sargodha, Sargodha 40100, Pakistan; Email: fqanwar@yahoo.com; 3 Department of Botany, University of Agriculture Faisalabad-38040, Pakistan; Email: ashrafbot@yahoo.com; 4 Department of Botany and Microbiology, King Saud University, Riyadh-11451, Saudi Arabia; 5 Institute of Tropical Agriculture, University Putra Malaysia, Serdang, Selangor 43400 UPM, Malaysia

**Keywords:** guava, ripening, vitamin C, phenolic contents, antioxidant activity

## Abstract

The present investigation was carried out to appraise the levels of total phenols and vitamin C as well as antioxidant potential at three different ripening stages (un-ripe, semi-ripe and fully-ripe) of guava (*Psidium guajava* L.) fruit collected from three different geographical regions of Pakistan (Islamabad, Faisalabad and Bhakkar). The antioxidant potential of guava fruit extracts was assessed by means of different *in-vitro* antioxidant assays, namely inhibition of peroxidation in linoleic acid system, reducing power and radical scavenging capability. Overall, fruit at the un-ripe stage (G1) exhibited the highest levels of TPC, TFC, reducing power and DPPH radical scavenging activity, followed by the semi-ripe (G2) and fully-ripe (G3) stages. On the other hand, vitamin C content increased as the fruit maturity progressed, with highest value seen at the fully-ripe stage (G3) followed by the semi-ripe (G2) and un-ripe stage (G1). The concentration of vitamin C in fruits varied as: Faisalabad (136.4–247.9 mg 100 g^−1^), Islamabad (89.7–149.7 mg 100 g^−1^) and Bhakkar (73.1–129.5 mg 100 g^−1^). The results showed that different stages of maturation and geographical locations had profound effects on the antioxidant activity and vitamin C contents of guava fruit.

## 1. Introduction

Fruits from many plant species contain a variety of antioxidant compounds such as vitamins, phenolics (flavonoids, phenolic acids), glutathione, and carotenoids. These antioxidants can act as decomposers of peroxides, scavengers of free radicals, quenchers of singlet and triplet oxygen, synergists and inhibitors of enzymes [1]. Besides, these antioxidant compounds possess a multitude of health functions including protection against cancer and coronary heart diseases. The protective effects of natural antioxidants are due to their considerable ability to retard or alleviate the extent of oxidative damage by reacting with free radicals [2].

Guava is a delicious fruit of the plant family Myrtaceae. Commonly known as Apple of Tropics it is a popular tree fruit of the Asian subcontinent [3]. The genus ‘*Psidium*’ comprises about 150 species, out of which the “Common Guava” (*Psidium* *guajava* L.), “Cattley guava” (*Psidium* *cattleianum* Sabine) “pear guava” (*Psidium* *pyriferum* L.) and “apple-guava” (*Psidium* *pomiferum* L.) are some of the important species. In Pakistan, the species *Psidium* *guajava* L., which yields about 100–300 fruit per tree, is widely cultivated for its delicious fruit [4].

Guava (*Psidium* *guajava* L.) is valued as a potential source of pectin, ascorbic acid (vitamin C), sugars and minerals [5]. Like other fruits and vegetables, guava is also a rich source of antioxidants and thus can help to prevent degenerative diseases. Different parts of guava have been traditionally used in the folk medicine of several civilizations [6]. The leaves have been extensively used for the treatment of diarrhea [7], bacterial infection, pain and inflammation [8]. An essential oil isolated from the leaves has shown anti-cancer properties [9]. The bark extract has long been in use for the treatment of diabetes [10]. 

It is generally considered that different parameters such as season, variety, stages of maturity and climatic conditions influence the phytochemical composition of fruits [11]. According to Yin and Chin [12] the extracts from guava (*Psidium guajava* L.) leaves exhibited good antioxidant activity as well as free radical-scavenging capacity. As far as we know there is no comprehensive data reported on the variation of antioxidants during development of guava fruit from different geographical regions of Pakistan. Therefore, the present research work aimed at evaluating and quantifying the vitamin C and other antioxidant attributes of guava fruits from different regions assayed at different stages of maturity.

## 2. Results and Discussion

### 2.1. Percentage Yield of Antioxidant Components from Guava Fruit

The results in Table 1 revealed that as the guava fruit maturity progressed, the percentage yields of methanol soluble extracts increased. The fully ripened fruits from the three regions selected gave the highest methanolic extract yields (18.92–24.91%), while those picked at the un-ripe stage offered the lowest (12.05–13.23%). The recovery of antioxidant components from plant materials mainly depends upon the amount, nature and the concentration of solvents used in an extraction method. Consequently, a suitable solvent system should be employed to extract optimum amount of antioxidant components. The extraction yield of such components generally increases by increasing the polarity of the solvents due to polar characteristics of antioxidants [13]. Methanol is often used for the extraction of antioxidant compounds from fruits. According to a previous investigation, fruits extract contained greater amounts of antioxidant compounds, when produced with methanol due to its high solubility potential [14].

**Table 1:** Percentage yield (g 100 g^−1^ dry wt.) of methanolic extracts from guava fruits collected at different stages of ripening.

Locality	G1 (un-ripe)	G2 (semi-ripe)	G3 (fully-ripe)
**Islamabad**	12.71 ± 0.13 ^B^_a_	13.82 ± 0.27 ^B^_b_	20.32 ± 0.17 ^A^_b_
**Faisalabad**	13.23 ± 0.11 ^C^_a_	15.17 ± 0.15 ^B^_a_	24.91 ± 0.54 ^A^_a_
**Bhakkar**	12.05 ± 0.23 ^B^_a_	13.78 ± 0.63 ^B^_b_	18.92 ± 0.77 ^A^_c_

Values are mean ± SD of three samples analyzed individually in triplicate; Superscripts within the same row represent significant variation (*p* < 0.05) among ripening stages; Subscripts within the same column represent significant variation (*p* < 0.05) among regions.

During the course of maturity and development, fruits undergo several changes in flavor, texture and color due to qualitative and quantitative variation in the composition of phytochemicals. Un-ripe fruits are usually hard in consistency, acidic in flavor, stiff and sometimes astringent [15]. They become sweet, soft, less astringent, less acidic, and well flavored as a result of ripening, thus becoming more palatable for human consumption. During ripening a series of complex biochemical reactions affect the formation of phenolics, anthocyanins, carotenoids and other volatile compounds leading to the development of final characteristics and distinct flavor of a mature fruit [16,17]. 

The results of the present study are in agreement to those reported by Wetwitayaklung *et al*. [18] who evaluated the compositional changes from the pulp and peel of Spanish plum fruits during maturation. The variation in the results of percentage yield of fruit extracts of guava from different locations may have been due to the influence of climatic changes on the chemical composition [19].

### 2.2. Total Phenolic Compounds

Total phenolic contents (TPC) of fresh guava fruit extracts were determined using the Folin-Ciocalteu method. This method was selected due to its practical applications and better sensitivity and promptness to determine the phenolics as compared to other tests in practice. The Folin-Ciocalteu reagent reacts preferably with phenolic compounds; however, it can be reduced by some non-phenolic compounds, e.g., Cu (II), vitamin C, *etc*. Although the precise response of the reagent with reducing group of species is not known, it is predicted that it forms a complex between phenolic ion (reducing species) and phosphomolybdic tungstate, changing the color from yellow to blue, the absorbance of which is measured at 755 nm.

The changes in TPC during maturation and ripening of guava fruit are presented in Table 2. In contrast to the extract yields, the fruits from the three selected locations contained higher concentrations of total phenolics at the un-ripe (G1) stage (24.81–33.16 mg GAE 100 g^−1^ extract dry wt.), while the minimum at the fully-ripened (G3) stage (11.47–30.22 mg GAE 100 g^−1^ extract dry wt.). The highest TPC values were determined in the fruit samples from Faisalabad, followed by Bhakkar and Islamabad, showing considerable variations among the regions. The fruit phenolic contents can be affected by many factors such as the variety, cultivation, species, area, ripeness, harvesting time, climatic conditions, storage time and environment [20,21]. The environmental factors are considered to play a major role on polyphenol content. These factors may be agronomic (biological culture, greenhouses or fields, hydroponic culture, fruit yield per tree, *etc*.) or climatic (sun exposure, soil type, rainfall). Besides, the concentration of polyphenols is also influenced by the extent of fruit’s maturity [22]. TPC of fully-ripen (mature) guava fruits from different geographical areas as determined in the present analysis were found to be lower than those reported elsewhere in four clones of guava fruit at maturity (170–344 mg GAE 100 g^−1^ extract) [23].

**Table 2:** Total phenolic contents (mg GAE 100 g^−1^ dry wt.) of extracts from guava fruit collected at different stages of ripening.

Locality	G1 (un-ripe)	G2 (semi-ripe)	G3 (fully-ripe)
**Islamabad**	24.81 ± 0.11 ^A^_b_	18.45 ± 0.63 ^B^_c_	11.47 ± 0.64 ^C^_c_
**Faisalabad**	32.72 ± 0.11 ^A^_a_	31.65 ± 0.15 ^A^_a_	30.22 ± 0.88 ^A^_a_
**Bhakkar**	33.16 ± 2.24 ^A^_a_	26.51 ± 2.72 ^B^_b_	20.54 ± 0.77 ^C^_b_

Values are mean ± SD of three samples analyzed individually in triplicate; Superscripts within the same row represent significant variation (*p* < 0.05) among ripening stages; Subscripts within the same column represent significant variation (*p* < 0.05) among regions.

The results of total phenolic contents reported in medlar fruit [24] also support our findings which showed that as the fruit ripening proceeded through ripe to over-ripe stage, an apparent gradual decrease was observed in the total fruit phenolic contents, which might be associated with an amplified polyphenol oxidase activity [25]. Another reason for the decrease in TPC might have been a loss in astringency during ripening, which may be associated with an increase in polymerization of leucoanthocyanidins and hydrolysis of the astergingent arabinose ester of hexahydrodiphenic acid [26].

In agreement to our observations, a decreasing trend in the phenolic contents during ripening was also reported in high bush blueberries [27,28]. The variations of TPC in the present analysis of guava fruits among different locations could be explained by the influence of temperature and different prevalent environmental factors [29].

### 2.3. Total Flavonoid Contents (TFC)

The results for the distribution of TFC in relation to fruit maturity/ripening stages and regions studied are presented in Table 3. 

**Table 3:** Total flavonoid contents (mg CE 100 g^−1^ dry wt.) of extracts from guava fruit collected at different stages of ripening.

Locality	G1 (un-ripe)	G2 (semi-ripe)	G3 (fully-ripe)
**Islamabad**	28.82 ± 0.67 ^A^_c_	24.61 ± 1.44 ^B^_b_	21.86 ±1.42 ^C^_b_
**Faisalabad**	46.08 ± 2.36 ^A^_a_	43.10 ± 0.73 ^B^_a_	31.09 ± 1.03 ^C^_a_
**Bhakkar**	35.05 ± 0.69 ^A^_b_	26.78 ± 0.90 ^B^_b_	18.65 ± 1.52 ^C^_c_

Values are mean ± SD of three samples analyzed individually in triplicate; Superscripts within the same row represent significant variation (*p* < 0.05) among ripening stages; Subscripts within the same column represent significant variation (*p* < 0.05) among regions.

The concentrations of flavonoids, with the exception of fruits from Islamabad, were generally higher in the extracts from un-ripe samples (G1: stage) as compared to those harvested at the semi-ripe (G2) and fully-ripe (G3) stages. The highest TFC were found in samples from Faisalabad (31.09–46.08 mg CE 100 g^−1^ dry wt.) followed by those from Bhakkar (18.65–35.05 mg CE 100 g^−1^ dry wt.) and Islamabad (21.86–28.82 mg CE 100 g^−1^ dry wt.). Overall, the magnitude of TFC in guava fruit with regard to the three different localities and maturity stages was found to decrease in the following order:

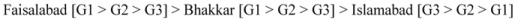


The higher concentrations of flavonoid compounds in younger (G1: un-ripe stage) fruit as compared to those in semi-ripe (G2) and fully-ripe (G3) fruits could be explained by the fact that in the later stages of ripening different phenolic acids might have condensed to form complex phenolic compounds such as tannins and lignin, *etc*. [30]. Hence, due to changes of phenolic compounds with maturity, fully-ripe fruit possessed relatively lower amounts of TF than those of semi-ripe and unripe fruits. 

Differences in TFC of different samples of guava at different stages of maturity could also be explained by an earlier report which stated that the presence of phenolics is influenced by growing conditions, genetic makeup of the species, soil circumstances and availability of nutrients at harvest level [31]. These factors might have affected the antioxidant activity of the fruits tested by affecting the composition of their phytonutrients. Moderate temperature conditions (25/30 °C) are suitable for increased antioxidant activity and total phenolic content. It has been reported that, plants grown in the cold (18/12 °C) or extreme hot (above 35 °C) temperature conditions, produce fruit which usually has lower antioxidant potential [29,32]. These findings supported our results which demonstrated that antioxidant activity of the fruit samples from moderately hot region (Faisalabad) was relatively higher than that of the fruits from Islamabad with a colder climate and Bhakkar with relatively higher temperature, suggesting that environmental factors have a significant influence on antioxidant potential. A similar decreasing trend in TFC was also observed in the bark, leaves, flower and pulp of fruit of *Cassia fistula* [33] as well as in blackberry, raspberry, and strawberry [20]. In agreement to our present trends, the earlier reported results [34] of total flavonoid contents of white tip fruit (unripe) compared with that of red tip fruit (ripe stage) also revealed that the flavonoid concentrations at the un-ripe stage (795 mg kg^−1^) were higher than those at the ripe stage (576 mg kg^−1^). 

### 2.4. DPPH^•^ Scavenging Activity

The relative antioxidant ability as measured by percent inhibition of DPPH^•^ by guava fruit extracts is shown in Table 4. Consistent with the trends for TPC, the methanolic extracts at concentration of 1.0 mg L^−1^ of dry matter of guava fruit from the selected three regions at un-ripe (G1) stage exhibited the highest DPPH scavenging capacity, *i.e*., (42.53–46.82%), while the lowest, *i.e*., 39.35–38.68%) was at the fully-ripe stage. Our findings are in agreement to those of Lim *et al*. [35] who reported that the greater DPPH scavenging activity of guava fruit at the un-ripe stage might be associated to its higher TPC levels rather than vitamin C ones.

**Table 4:** DPPH radical scavenging activity of extracts from guava fruit at different stages of ripening.

Locality	Ripening stages	Concentration of extract (mg L^−1^)		
		0.01	0.1	1.0
**Islamabad**	**G1(un-ripe)**	34.06 ± 0.15	38.94 ± 0.20	42.53 ± 0.32
	**G2(semi-ripe)**	32.84 ± 0.09	37.87 ± 0.22	39.60 ± 0.11
	**G3(fully-ripe)**	31.84 ± 0.12	33.58 ± 0.19	39.35 ± 0.18
**Faisalabad**	**G1(un-ripe)**	39.64 ± 0.23	40.09 ± 0.17	46.82 ± 0.28
	**G2(semi-ripe)**	30.45 ± 0.29	35.25 ± 0.24	45.84 ± 0.33
	**G3(fully-ripe)**	21.56 ± 0.34	32.73 ± 0.40	38.25 ± 0.31
**Bhakkar**	**G1(un-ripe)**	32.62 ± 0.24	34.07 ± 0.19	43.72 ± 0.21
	**G2(semi-ripe)**	24.33 ± 0.15	31.87 ± 0.11	43.51 ± 0.18
	**G3(fully-ripe)**	23.88 ± 0.22	31.85 ± 0.21	38.68 ± 0.34

Values are mean ± SD of three samples analyzed individually in triplicate.

Of the regions studied, the extracts from guava fruit from Faisalabad had higher free radical scavenging activity, followed by those from Bhakkar and Islamabad. The process of lipid peroxidation entails the formation of free radicals which are considered to play a key role in various persistent pathologies, such as cardiovascular diseases as well as cancer [36,37]. A compound possessing radical reducing power can act as a potential antioxidant to reduce the incidence of such diseases [38,39].

The DPPH free radical scavenging capacity of guava fruit extracts increased in a concentration dependent manner (Table 4). The DPPH^•^ is decolorized by accepting an electron donated by an antioxidant. The reducing potential of a substrate usually depends on the concentration of reductants [40], which exhibit antioxidant activity by donating a hydrogen atom and breaking the chain of free radicals. The increases in concentration of antioxidants is linked to increasing the scavenging of DPPH^•^ and thus an indication of higher antioxidant activity [41].

The present decreasing trend of DPPH^•^ free radical scavenging activity is in line with the investigation of Kulkarni and Aradhya [42] who reported a decrease in antioxidant activity of pomegranate arils by 13% from 20 to 60 days of fruit development. The decline in scavenging capacity during maturation might be linked to decrease in the concentration of total phenolics, and rapid consumption of anthocyanins and compositional changes as a result of fruit development [16].

As far as variation in the results of different locations is concerned, Bhakkar guava fruit samples showed the highest values, whereas those from Islamabad exhibited the lowest antioxidant activity, which is in agreement with the findings of Connor *et al*. [43] who found that antioxidant content varied significantly in blueberry fruit harvested from different locations and in different years. This may reflect differences in cultural practices and climatic conditions among locations, including differences in ultraviolet radiation, temperature, or water stress, or mineral nutrient availability. The soil types as well as fertilization parameter also influence the nutritional composition and antioxidant activity by affecting the water and nutrient supply to the plant of the harvested fruit [29].

### 2.5. Reducing Power of Guava Fruit Extracts

The reducing power of extracts of guava fruit during ripening was determined according to the method reported by Yen *et al*. [44] with slight modifications. The results for the reducing potential of guava fruit extracts derived at different ripening stages are shown in Table 5.

**Table 5:** Reducing potential (absorbance at 700 nm) of extracts from guava fruit at different stages of ripening.

Locality	Ripening stages	Concentration of guava fruit extract (mg·L^−1^)			
		2.5	5.0	7.5	10.0
**Islamabad**	**G1(un-ripe)**	1.18 ± 0.19	1.45 ± 0.39	1.66 ± 0.22	1.86 ± 0.21
	**G2(semi-ripe)**	0.92 ± 0.24	1.42 ± 0.24	1.52 ± 0.23	1.77 ± 0.26
	**G3(fully-ripe)**	0.80 ± 0.72	1.38 ± 0.28	1.45 ± 0.14	1.71 ± 0.09
**Faisalabad**	**G1(un-ripe)**	1.65 ± 0.21	1.89 ± 0.23	1.93 ± 0.35	1.95 ± 0.19
	**G2(semi-ripe)**	1.65 ± 0.18	1.84 ± 0.19	1.85 ± 0.16	1.92 ± 0.23
	**G3(fully-ripe)**	1.20 ± 0.24	1.43 ± 0.31	1.64 ± 0.21	1.89 ± 0.30
**Bhakkar**	**G1(un-ripe)**	1.12 ± 0.27	1.64 ± 0.27	1.79 ± 0.13	1.91 ± 0.18
	**G2(semi-ripe)**	1.07 ± 0.27	1.63 ± 0.36	1.77 ± 0.22	1.88 ± 0.19
	**G3(fully-ripe)**	0.94 ± 0.17	1.38 ± 0.33	1.74 ± 0.25	1.84 ± 0.25

Values are mean ± SD of three samples analyzed individually in triplicate.

The reducing potential of guava fruit extracts, measured at the concentrations of 2.5, 5.0, 7.5, 10 mg·L^−1^ were found to be increased in a concentration dependent manner (Table 5), at un-ripe (G1) stage guava fruit samples exhibited the highest reducing potential (1.86, 1.95, 1.91), followed by those of the semi-ripe (G2) stage (1.77, 1.92, 1.88) and fully-ripe (G3) stage (1.77, 1.89, 1.84). In relation to the regions and the fruit maturity stages, the reducing potential of guava fruit was observed to decrease in the following order:

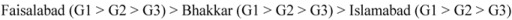


Such a decline in reducing power as maturity progressed might be associated with the trends of the results for total phenolics in this analysis. As the phenolics are considered to be very effective reducing agents and therefore the association between their contents and the decreasing reducing potential of the tested fruit extracts is rather understandable [21].

Siddhuraju *et al*. [33] and Yildirim *et al*. [45] also investigated the direct relation of antioxidant activity with the reducing power of bioactive compounds. The highest reducing potential in the later stage of maturation of orange pulp in “Red flash variety”, but decreasing trend in case of “Newhall variety” was reported by Huang *et al*. [46], revealing that antioxidant activity was not consistent; it may depend upon cultivar and fruit ripening stage. It has also been reported that the antioxidant activity of fruits could be affected by harvest time, storage time, geographical origin or cultivar [47], temperature, storage [48,49] or use of exogenous chemicals [50].

### 2.6. Antioxidant Activity by Inhibition of Linoleic Acid Peroxidation

Inhibition of linoleic acid oxidation showed significant (*p* < 0.05) differences for analyzed extracts of guava fruit during the ripening process. It is evident from Table 6 that methanolic extract of guava fruit at un-ripe stage (G1) from Faisalabad exhibited the highest inhibition of linoleic acid peroxidation and thus reflected the strongest antioxidant activity (97.05%), followed by that of the samples from Bhakkar (92.63%) and Islamabad (76.70%). Among the regions and ripening stages selected, the magnitude of inhibition of linoleic acid peroxidation of the tested fruits decreased in the following order:

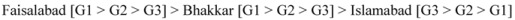


**Table 6:** Percentage inhibition of linoleic acid peroxidation of extracts of guava fruit at different stages of ripening.

Locality	G1 (un-ripe)	G2 (semi-ripe)	G3 (fully-ripe)
**Islamabad**	76.70 ± 0.19 ^A^_c_	60.94 ± 0.40 ^B^_c_	45.35 ± 0.44 ^C^_c_
**Faisalabad**	97.05 ± 0.11 ^A^_a_	88.23 ± 0.24 ^B^_a_	86.76 ± 0.37 ^C^_a_
**Bhakkar**	92.63 ± 0.23 ^A^_b_	64.59 ± 0.09 ^B^_b_	54.41 ± 0.32 ^C^_b_

Values are mean ± SD of three samples analyzed individually in triplicate; Superscripts within the row represent significant variation (*p* < 0.05) among ripening stages; Subscripts within the column represent significant variation (*p* < 0.05) among localities.

The inhibition of linoleic acid oxidation was also employed to assess the antioxidant potential of the guava fruit extracts. Antioxidant activity of different extracts of guava fruit during maturation was determined by inhibition of peroxidation in linoleic acid system using the thiocyanate method [51]. During the process of linoleic acid peroxidation, the peroxides were formed and as a result, the compounds oxidized Fe^2+^ to Fe^3+^. The Fe^3+^ ions then formed a complex with SCN^−^, which had highest absorbance at 500 nm. It is the most widely used method for evaluation of antioxidant potential and for determining the inhibitory extent of auto-oxidation of linoleic acid.

We can conclude from this assay that fresh newly growing fruit of guava shows the highest antioxidant potential. The variation in the results of different samples from different localities are comparable with the findings of Iqbal and Bhanger [21], who showed that due to high temperature conditions the samples from hot regions exhibit the least antioxidant activity. These results support our findings as Faisalabad experiences moderate temperature conditions as compared to Bhakkar (hot) and Islamabad (cold) and its soil is more fertile than those of the other two localities. There is no earlier report available on the inhibition of linoleic acid oxidation of guava fruit extracts with which our present results can be compared. 

### 2.7. Vitamin C Contents of Guava Fruit at Different Stages of Maturation

Vitamin C content of the fresh fruits of guava fruit was estimated by the method as described by Thaipong *et al*. [23]. The results shown in Table 7 indicate an increasing trend of vitamin C contents with fruit ripening. It is evident that at the fully-ripe stage (G3) guava fruit from Faisalabad exhibited the highest vitamin C (247.93 mg 100 g^−1^) contents. The overall trend of vitamin C contents (100 mg g^−1^) of guava fruit samples among different regions during maturation was observed to be:

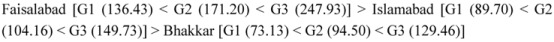


**Table 7:** Vitamin C contents (mg 100 g^−1^) of guava fruit at different stages of ripening.

Locality	G1 (un-ripe)	G2 (semi-ripe)	G3 (fully-ripe)
**Islamabad**	89.70 ± 0.19 ^C^_c_	104.16 ± 1.02 ^B^_b_	149.73 ± 0.26 ^A^_b_
**Faisalabad**	136.43 ± 1.71 ^C^_a_	171.20 ± 0.28 ^B^_a_	247.93 ± 1.42 ^A^_a_
**Bhakkar**	73.13 ± 1.32 ^B^_b_	94.50 ± 1.03 ^B^_b_	129.46 ± 1.22 ^A^_c_

Values are mean ± SD of three samples analyzed individually in triplicate; Superscripts within the row represent significant variation (*p* < 0.05) among ripening stages; Subscripts within the column represent significant variation (*p* < 0.05) among localities.

Vitamin C, also known as ascorbic acid (AA), is considered as an enzymatic cofactor. It plays a key role as an essential compound for plant tissues due to its considerable antioxidant role [52]. In fruits, variation in vitamin C contents due to several factors such as variety, species, cultivation practice and harvesting conditions has been reported. The other variables such as ambient temperature, photosynthetic process, relative humidity, oxidative stress, exposure of sun as well as pollutants are also considered as main contributors responsible for the variation in vitamin C contents. Different fruits exhibit different pattern of variation during storage and ripening processes. During the course of fruit ripening, vitamin C contents may decrease, increase or remain constant [53].

The results obtained are comparable with those reported by Soares *et al*. [54], who showed an increasing trend in vitamin C contents with maturation. According to their study in immature fruit the amount of vitamin C was 76.8 mg 100 g^−1^ of sample, and it became 126.21 and 168.36 mg 100 g^−1^ at the mature and fully ripe stages, respectively. The increase in vitamin C contents of guava fruit with progress in maturity might have been due to the breakdown of starch to glucose which increases the biosynthesis of ascorbic acid [35].

An increase in vitamin C content as the fruit matured (32 mg 100 g^−1^ to 144 mg 100 g^−1^ sample) was also reported by Lim *et al*. [35]. Gomez and Lajolo [52] exhibited 50% increase in vitamin C contents as a result of maturation in case of guava, but 35% decrease in vitamin C contents in case of mango during ripening. This inconsistent behavior of some fruits and different cultivars might be due to geographical and environmental conditions such as rain, temperature and soil [55].

## 3. Experimental

### 3.1. Materials

#### 3.1.1. Samples

The samples of pear-shaped fruits of guava (*Psidium guajava* L.) were collected from three different trees from three orchards in each of the three geographical regions of Pakistan, namely Islamabad, Bhakkar and Faisalabad, in the month of October 2010. These areas belong to well established irrigated regions. Islamabad is situated at 33°40'N latitude and 73°10'E longitude and lies at the edge of Pothohar plateau south of Margla hills, with an average humidity level and average max/min air temperatures of 59% and 33/21 °C, respectively; Faisalabad stands in the rolling smooth plains of northeast Punjab, between latitude 30°31.5'N and longitude 73°74'E, humidity level and average max/min air temperature 56% and 35/24 °C, respectively, while Bhakkar District comprises plains and deserts situated at latitude 31°38'00"N and longitude 71°04'00"E with humidity level and average max/min air temperature, 62% and 33/18 °C, respectively. Guava fruits (one kg for each sample) were picked at each ripening stage; at the un-ripen stage (G1), only fully green hard fruits; at the semi-ripen stage (G2), slightly greenish yellow somewhat firm, while at the fully-ripen stage (G3) yellow and soft fruits were picked. The fresh guava fruits were washed with distilled water and finely chopped for obtaining the extracts.

#### 3.1.2. Chemicals and Reagents

The HPLC grade standard of ascorbic acid, linoleic acid (±), catechin, gallic acid and Folin-Ciocalteu reagent were purchased from the Sigma Chemical Co. (St. Louis, MO, USA). All other chemicals including acetonitrile, methanol, and acetic acid used in this study were procured from Merck (Darmstadt, Germany).

### 3.2. Methods

#### 3.2.1. Extraction

Chopped samples (20 g each) of fresh guava (*Psidium guajava* L.) were extracted with 80% methanol (80:20 methanol-water v/v, 200 mL) in 500 mL conical flasks and shaken for 24 h at room temperature in an orbital shaker (Gallenkamp, Loughborough, UK). All extracts were separated from the residues by filtering through Whatman No.1 filter paper. The residues were extracted twice in the same manner and the extracts combined. The combined extracts were concentrated and freed of solvent under reduced pressure at 45 °C, using a rotary evaporator (EYELA, SB-651, Rikakikai Co. Ltd. Tokyo, Japan). The crude concentrated extracts were weighed to calculate the yield and then stored at −4 °C until used for further analysis.

#### 3.2.2. Estimation of Total Phenolic Contents (TPC)

The total phenolic compounds were determined by Folin-Ciocalteu method [56]. Standard gallic acid solutions with varying concentrations ranging from 0.01−0.12 mg·mL^−1^ in methanol were prepared for calibration purposes. The absorbance was noted after one hr at 765 nm and the calibration curve plotted by taking absorbance as a function of concentration. One mL of guava extract (10 g·L^−1^) was mixed with the same reagent as mentioned above and after one hr the absorbance of the resulting blue colored solution was measured at 765 nm with a UV visible spectrophotometer (U-2001, HitachiInstruments Inc., Tokyo, Japan). Quantification was done with respect to the standard. All determinations were performed in triplicate. 

#### 3.2.3. Determination of Total Flavonoid Contents (TFC)

The TFC were measured by a spectrophotometric method previously reported by Dewanto *et al.* [57]. One mL of the aqueous extract containing 0.1 g/mL of extract was placed in a 10 mL volumetric flask, then distilled water (5 mL) was added, followed by 5% NaNO_2_ (0.3 mL). After 5 min, 10% AlCl_3_ (0.6 mL) was added to the mixture. After another 5 min, 1 M NaOH (2 mL) was added and the volume made up with distilled water. The solution was mixed and absorbance read at 510 nm with a UV-visible spectrophotometer (U-2001, Hitachi Instruments Inc., Tokyo, Japan). TF concentrations were expressed as catechin equivalents on dry weight basis. All samples were analyzed thrice and results averaged.

#### 3.2.4. DPPH Scavenging Activity

The DPPH assay was performed as described by Bozin *et al*. [58]. Samples ranging from 0.2 to 500 µg·mL^−1^ were mixed with 90 µM DPPH solution (1 mL) and filled up with 95% methanol, to a final volume of 4 mL. The absorbance of the resulting solutions and the blank were recorded after 1 h at room temperature. Butylated hydroxytoluene (BHT) was used as a positive control. Data for three replicates within each sample were recorded. The disappearance of DPPH was examined spectrophotometrically at 515 nm using a spectrophotometer (U-2001, Hitachi Instruments Inc., Tokyo, Japan). Inhibition of free radical (DPPH**^•^**) in percent (%) was calculated in the following way:

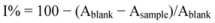

where A_blank_ is the absorbance of the control reaction mixture excluding the test compounds, and A_sample_ is the absorbance of the test compounds. 

#### 3.2.5. Determination of Reducing Power

The reducing power of each extract was investigated according to the procedure described by Yen *et al*. [44] with slight modifications. Concentrated extract (2.5–10.0 mg) was mixed with sodium phosphate buffer (5.0 mL, 0.2 M, pH 6.6) and potassium ferricyanide (5.0 mL, 1.0%); the mixture was incubated at 50 °C for 20 min. Then 10% trichloroacetic acid (5 mL) was added and the mixture centrifuged at 980 g for 10 min at 5 °C in a refrigerated centrifuge (CHM-17; Kokusan Denki, Tokyo, Japan). The upper layer of the solution (5.0 mL) was decanted and diluted with distilled water (5.0 mL) and 0.1% ferric chloride (1.0 mL,), and the absorbance read at 700 nm using spectrophotometer (U-2001, Hitachi Instruments Inc., Tokyo, Japan). All samples were analyzed thrice and results averaged.

#### 3.2.6. Determination of Antioxidant Activity by Linoleic Acid Peroxidation

The antioxidant activity of the tested guava extracts was also determined by inhibiting the oxidation of linoleic acid [51]. Five mg of each extract were added separately to a solution of linoleic acid (0.13 mL), 99.8% ethanol (10 mL) and 0.2 M sodium phosphate buffer (10 mL, pH = 7). The mixture was made up to 25 mL with distilled water and incubated at 40 °C up to 360 h. The extent of oxidation was measured by peroxide value using the thiocyanate method as described by Yen *et al*. [44]. Briefly, ethanol (10 mL, 75% v/v), aqueous solution of ammonium thiocyanate (0.2 mL, 30% w/v), sample solution (0.2 mL) and ferrous chloride (FeCl_2_) solution (0.2 mL, 20 mM in 3.5% HCl; v/v) added sequentially. After 3 min of stirring, the absorption was measured at 500 nm using a UV-Visible spectrophotometer (U-2001, Hitachi Instruments Inc., Tokyo, Japan). A control contained all reagents excluding the extracts was used. Synthetic antioxidant butylated hydroxytoluene (BHT) was used as positive control. Percentage inhibition of linoleic acid peroxidation was calculated with the help of the following equation: 100 − [(Abs. increase of sample at 360 h/Abs. increase of control at 360 h) × 100], to express the antioxidant activity.

#### 3.2.7. Determination of Vitamin C Contents by HPLC

Vitamin C (ascorbic acid) content of each sample extract was investigated according to the procedure described by Thaipong *et al*. [23]. Fruit extracts for ascorbic acid analysis were obtained by homogenizing guava tissue (pulp and peel, 3 g) in cold solution of 3% (w/v) oxalic acid + 8% glacial acetic acid (v/v) (20 mL) until a uniform consistency using an Ultra-Turrax homogenizer (T25, Ika Works Inc., Houston, TX, USA). The homogenates were centrifuged at 15,000 rpm at 4 °C for 10 min. The supernatants were recovered and the amount of vitamin C was obtained by HPLC using a fluorescence detector (UV). The column was shim-pack CLC-ODS (C18), 15 cm × 4.6 mm, 5 µm. The chromatographic conditions were: wavelengths of 325 nm and flow rate 1 mL·min^−1^. The mobile phase consisted of methanol and acetonitrile (50:50). The results of ascorbic acid were expressed as mg 100 g^−1^ of dry weight of guava fruit.

#### 3.2.8. Statistical Analysis

Three samples at each development stage from each location were assayed. Each sample was analyzed individually in triplicate and data were reported as mean (*n* = 3 × 3) ± SD (*n* = 3 × 3). Data were analyzed using two-way analysis of variance ANOVA using Minitab 2000 Version 13.2 statistical software (Minitab Inc., Centre County, PA, USA) at 5% significance level (*p* < 0.05).

## 4. Conclusions

From the present work, it could be concluded that geographical conditions and fruit ripening stage had profound effects on vitamin C contents and antioxidant activity of guava fruit. Antioxidant activity of samples from the moderate temperature region was relatively higher than those from cold or hot areas. These findings propose that temperature, soil type, availability of nutrients and other environmental factors have a significant effect on the antioxidant potential of guava fruit. Proper agro-climatic and harvesting regimes should be sought to obtain the maximum nutritional and medicinal benefits of such fruits.
